# E-selectin, but not CRP, partially mediates the association between metabolic indices and insulin resistance in older adults: a mediation analysis

**DOI:** 10.2478/abm-2026-0007

**Published:** 2026-04-30

**Authors:** Laith Ashour, Tariq Albweitel, Juana Abu Rahmeh, Momen Alfawadleh, Afaf El Sharabi, Rahmah Ahmad Shareah, Zaid Alomari, Arar Alhawwari, Mohammad Alkalouti, Noura F. Al-Nawaiseh, Malak S. Ababneh, Abdelrahman Kaoukji

**Affiliations:** Public Health Institute, Liverpool John Moores University, Liverpool L2 2QP, UK; Department of Medicine, Faculty of Medicine, University of Jordan, Amman 11942, Jordan; Prince Hamza Hospital, Amman 11947, Jordan; Jordan University Hospital, University of Jordan, Amman 11942, Jordan; Department of Medicine, Faculty of Medicine, Yarmouk University, Irbid 21163, Jordan; Independent Medical Researcher and Practitioner, Amman, Jordan; King Hussein Medical Center, Jordanian Royal Medical Services, Amman 11855, Jordan; Department of Medicine, Faculty of Medicine, Al-Balqa Applied University, Al-Salt 19117, Jordan; Ministry of Health, Amman 11118, Jordan; Department of Medicine, Specialty Hospital, Amman 11193, Jordan

**Keywords:** C-reactive protein, E-selectin, insulin resistance, metabolic indices, older adults

## Abstract

**Background:**

Evidence on the pathophysiology of insulin resistance (IR), particularly the mediating role of inflammatory markers, remains limited.

**Objectives:**

Prior studies suggest associations of C-reactive protein (CRP) and E-selectin with IR, generally examined independently of other risk factors. This study evaluates their potential mediation roles.

**Methods:**

Using biomarker data from the Midlife in the United States 3 (MIDUS3) 2017–2022 cohort, we conducted cross-sectional bias-corrected bootstrapping mediation analyses to assess whether CRP and E-selectin mediate associations between established risk factors—body mass index (BMI), waist-hip ratio (WHR), total cholesterol/HDL ratio, age, glycated hemoglobin A (HbA1c), smoking status, dietary habits, physical activity, medication use, and sex—and IR measured by log-transformed homeostatic model assessment of IR (HOMA-IR).

**Results:**

The study included 708 participants (57% female; mean age 66.2 ± 9.65 years). CRP did not mediate associations between covariates and HOMA-IR. In contrast, E-selectin showed indirect-only mediation for sex and HOMA-IR (% change in HOMA-IR [%CHIR] in females = 2.4; 95% CI: [0.46, 5.80]). Partial mediation by E-selectin was observed for HbA1c (%CHIR = 9.26; 95% CI: [3.40, 18.66]), BMI (%CHIR = 2.72; 95% CI: [0.97, 5.54]), and total/HDL cholesterol ratio (%CHIR = 1.92; 95% CI: [0.34, 4.49]). Indirect-only mediation was also found for age (%CHIR = −1.11; 95% CI: [−2.58, −0.27]), non-smoking (%CHIR = −1.55; 95% CI: [−4.09, −0.13]), and higher healthy eating index score (%CHIR = −7.87; 95% CI: [−17.48, −2.43]).

**Conclusion:**

E-selectin, but not CRP, mediates relationships between metabolic risk factors and IR, highlighting endothelial dysfunction as a key pathway

Insulin resistance (IR) is a metabolic condition characterized by impaired cellular response to insulin in target tissues, including skeletal muscle, liver, and adipose tissue [1]. It represents an early pathogenic driver for multiple conditions, including type 2 diabetes, metabolic syndrome, and atherosclerosis [1, 2]. The prevalence of IR increases with age, affecting over 40% of US adults aged 60 years and older [3]. In elderly individuals, IR is multifactorial: aging contributes to visceral fat accumulation, oxidative stress, and mitochondrial dysfunction, while excess adiposity, particularly abdominal fat, further elevates risk [4]. Dietary habits, physical inactivity, and loss of muscle mass also play critical roles [5,6,7].

IR has well-documented consequences in the elderly, contributing to a wide range of comorbidities, including worsened prognosis in acute coronary syndrome [8], metabolic-associated steatotic liver disease/non-alcoholic fatty liver disease (MASLD/NAFLD) [9], heart failure [10], dementia [11], arthritis [12], and psoriasis in postmenopausal women via shared genetic susceptibility, immune dysregulation, and systemic inflammation [13].

The pathophysiology underlying IR-related comorbidities involves chronic low-grade inflammation, driven primarily by cytokines secreted by resident macrophages and adipose tissue. These cytokines activate stress kinases such as c-Jun N-terminal kinase (JNK) and Inhibitor of kappa B kinase beta/Nuclear factor kappa-light-chain-enhancer of activated B cells (IKKβ/NF-κB), impairing insulin receptor signaling through serine phosphorylation of Insulin receptor substrate 1 and 2 (IRS-1/2) and inhibition of the Phosphoinositide 3-kinase (PI3K)/protein kinase B (Akt)/endothelial nitric oxide synthase (eNOS) pathway (PI3K/Akt/eNOS), thereby reducing nitric oxide (NO) bioavailability and altering endothelial function [14, 15]. Endothelial dysfunction, particularly at the arteriolar and capillary level, may exacerbate IR [16]. Consequently, levels of soluble E-selectin, an endothelial adhesion molecule, rise in response to metabolic and inflammatory disturbances [17, 18].

Chronic inflammation driven by metabolic risk factors also increases pro-inflammatory adipokines, including tumor necrosis factor-alpha (TNF-α) and interleukin-6 (IL-6), which upregulate hepatic C-reactive protein (CRP) synthesis [19]. Experimental evidence in rodents suggests that CRP can directly induce hepatic IR via Extracellular signal-regulated kinases 1 and 2 (ERK1/2) activation, impairing insulin signaling [20]. Clinical studies support these findings as Chinese patients with higher body mass index (BMI) and waist circumference had significantly higher circulating E-selectin and CRP levels, linking low-grade inflammation to obesity-related endothelial dysfunction [21].

Despite evidence that CRP and E-selectin are associated with IR, the specific factors influencing their levels remain unclear. Mediation analysis can clarify whether these biomarkers transmit the effects of metabolic and lifestyle risk factors on IR. To date, no studies have addressed this question. Therefore, the present study aims to determine whether E-selectin and CRP mediate associations between established risk factors and IR in elderly adults. We hypothesize that these 2 inflammatory markers mediate the association between metabolic factors and IR in older adults.

## Methods

### Study population

The Midlife in the United States (MIDUS) study is a longitudinal survey initiated in 1995, assessing psychological, behavioral, and social determinants of health in over 7,000 Americans aged 25–75 years, with oversampling in 5 metropolitan areas. Follow-up waves included MIDUS2 (2009) and MIDUS3 (2013), both incorporating biomarker data collection. The MIDUS3 biomarker project (2017–2022) involved a 24 h in-person assessment at one of three research centers (UCLA, University of Wisconsin-Madison, Georgetown University), collecting data on musculoskeletal, neurological, immune, and other system functions.

The pooled MIDUS3 biomarker sample included 747 participants (longitudinal survey: n = 644; Milwaukee sample: n = 103), with an overall adjusted response rate of 64.3% (747/1162).

In our analysis, due to the presence of missing cases in medication's use data, the total number of valid cases included in our study was 708.

### Study design and variables

This cross-sectional study utilized data exclusively from the MIDUS3 Biomarker 2017–2022 dataset.

**Figure 1.**
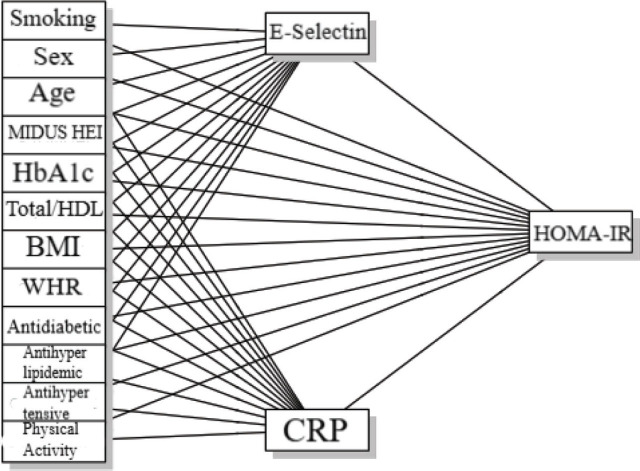
Conceptual path diagram illustrating the mediation model examining predictors of IR, with CRP and E-selectin as mediating variables. BMI, body mass index; CRP, C-reactive protein; HbA1c, glycated hemoglobin A; HOMA-IR, homeostatic model assessment of IR; IR, insulin resistance; MIDUS HEI, MIDUS healthy eating index; WHR, waist-hip ratio.

The independent variables selected to predict homeostatic model assessment of IR (HOMA-IR) in this study included history of regular smoking (yes/no), BMI, sex, total cholesterol/HDL ratio (as a measure of dyslipidemia), age, waist-hip ratio (WHR), MIDUS healthy eating index (HEI), glycated hemoglobin A (HbA1c), and physical activity (measured via Total number of metabolic equivalent of task [MET] minutes per week). We also controlled for medication use, specifically anti-diabetic, anti-hyperlipidemic, and anti-hypertensive medication use. The mediators of interest were CRP and serum soluble E-selectin. The dependent variable was IR, measured using the HOMA-IR index, which was calculated within the MIDUS dataset and required no additional computation by the authors (**Figure 1**).

Dietary habits were assessed in MIDUS using a questionnaire capturing the frequency of consumption of healthy foods (e.g., vegetables, fruits, lean meats, fish, whole grains) and unhealthy foods (e.g., fast food, fatty foods, sugar-sweetened beverages) weekly. Higher frequencies of healthy food consumption or lower frequencies of unhealthy food consumption were coded to yield higher scores for each food type. These scores were summed to generate the MIDUS HEI, which ranges from 0 to 11, with higher scores reflecting healthier dietary habits. This raw HEI score was incorporated directly into our analysis.

Additional details regarding the MIDUS3 study procedures, including the collection of serum biomarkers, are available on the study website and documentation [22].

### Statistical analysis

Descriptive statistics were used to summarize the basic characteristics of the study population. Continuous variables were reported as mean and standard deviation (SD) for normally distributed variables and median with interquartile range (IQR) for the non-normal ones, while categorical variables were summarized as counts and percentages.

To evaluate the potential mediation roles of E-selectin and CRP in the associations between the selected covariates and HOMA-IR, a general linear mediation model was employed. Given the right-skewed distribution of HOMA-IR values, log-transformation was applied to meet model assumptions. Coefficients obtained from the mediation analysis were subsequently back-transformed and expressed as percentage changes in HOMA-IR (%CHIR) for clinical interpretability. Percentage changes were reported per clinically relevant increments of the covariates: 10-year increase in age, 10-unit increase in HEI, 0.1-unit increase in WHR, 2-unit increase in total/high-density lipoprotein (HDL) cholesterol ratio, 3% increase in HbA1c, 1,000 min increase in number of MET, and 10 kg/m² increase in BMI. For the mediator variables, changes were presented per 10-unit increase in CRP and 10-unit increase in E-selectin.

Confidence intervals (CIs) were calculated using a bias-corrected bootstrapping method with 3,000 replications, providing robust estimation of 95% CIs. Missing data (after removal of data not missed at random from medication variables) were handled using full information maximum likelihood method, and continuous covariates were mean-centered to enhance the validity of results by avoiding multicollinearity. All predictors were modeled simultaneously in an adjusted analysis, and the analysis statistical assumptions were met after using bias-corrected bootstrapping. Log-transformation of the dependent variable was efficient to support the analysis assumptions, to handle any outliers, and to meet the linearity and other assumptions. Of note, all potential confounding variables were considered in the analysis.

For mediation paths, statistical significance was indicated when the 95% CI did not include zero (equivalent to *P* < 0.05). This approach aligns with contemporary recommendations for reporting bootstrapping-based mediation results rather than relying on *P-values*, which may be biased in this setting. All analyses were conducted using Jamovi software (The Jamovi Project, 2024; Version 2.6.13, https://www.jamovi.org).

This study is a secondary analysis of a publicly available dataset from the MIDUS study, ethical approval is waived. All MIDUS original study protocols were reviewed and approved by the University of Wisconsin-Madison Institutional Review Board.

## Results

The total number of valid cases was 708, with minor female predominance (57%). Mean age was 66.2 years (SD = 9.65 years). Notably, HOMA-IR values had a median of 2.47 (IQR: 1.44–4.32), indicating borderline IR in large proportion of sample. The geometric (i.e., back-transformed or exponentiated value) mean of HOMA-IR was 2.66 as shown in **Table 1**.

**Table 1.** Participant characteristics

**Characteristics**	**N**	**Total (%)**
Sex		
Male	307	43
Female	401	57
History of regular smoking		
Yes	309	44
No	399	56
Anti-diabetic use		
Yes	126	18
No	582	82
Anti-hyperlipidemic use		
Yes	328	46
No	380	54
Anti-hypertensive use		
Yes	365	52
No	343	48

**Descriptives of continuous basic characteristics**		
	**Mean**	**SD**
Log(HOMA-IR)	0.98	0.88
Age (years)	66.24	9.65
MIDUS HEI	5.47	1.49
Ratio total/HDL cholesterol	3.53	1.14
BMI (kg/m^2^)	29.93	6.59
WHR	0.91	0.1

	**Median**	**25th Percentile**	**75th Percentile**
Number of MET (min/week)^†^	666	0	1600
HbA1c (%)^†^	5.6	5.3	6
HOMA-IR^†^	2.47	1.44	4.32
Serum soluble E-selectin (ng/mL)^†^	33.35	23.77	46.07
CRP (μg/mL)^†^	1.79	0.78	4.6

† Not normally distributed.

BMI, body mass index; CRP, C-reactive protein; HbA1c, glycated hemoglobin A; HOMA-IR, homeostatic model assessment of IR; MET, metabolic equivalent of task; MIDUS HEI, healthy eating index; SD, standard deviation; WHR, waist-hip ratio.

As a reminder for interpreting mediation analysis results, the indirect effect represents the impact of an independent variable on Log-HOMAIR that is mediated by E-selectin or CRP. The direct effect represents the influence of the independent variable on Log-HOMAIR that occurs independently of the mediator (i.e., while holding the mediator constant).

Mediation analysis suggested that E-selectin mediated (indirect-only mediation) the positive association of female sex with HOMA-IR, as the indirect path was the only significant path (% change in HOMA-IR [%CHIR] = 2.4; 95% CI: [0.46, 5.80]). A similar pattern was observed among participants not taking antihypertensive medications ([%CHIR] = −2.39; 95% CI: [−5.25, −0.70]), likely due to the underlying hypertension in medication users, which contributes to endothelial dysfunction that may exacerbate IR. However, the association was partially mediated by E-selectin in cases of HbA1c (%CHIR = 9.26; 95% CI: [3.40, 18.66]), BMI (%CHIR = 2.72; 95% CI: [0.97, 5.54]), and total/HDL cholesterol ratio (%CHIR = 1.92; 95% CI: [0.34, 4.49]). These findings indicate that higher levels of these metabolic indices are associated with increased E-selectin, which in turn contributes to elevated HOMA-IR.

In contrast, E-selectin had an indirect-only negative mediation for associations between age (%CHIR = −1.11; 95% CI: [−2.58, −0.27]), non-smoking status (%CHIR = −1.55; 95% CI: [−4.09, −0.13]), and higher HEI score (%CHIR = −7.87; 95% CI: [−17.48, −2.43]) with HOMA-IR. This suggests that older age, healthier dietary habits, and absence of regular smoking may be associated with lower E-selectin levels, which subsequently reduce HOMA-IR. Indirect-only mediation was suggested by the lack of significant total and direct effects for these 3 variables (**Table 2**).

**Table 2.** Results of mediation analysis for the mediation of CRP and E-selectin for associations between metabolic factors and basic characteristics and HOMAR-IR in American older adults

**Type**	**Effect**	**95% CI^‡^**			
		**% change in HOMA IR^†^**	**Lower**	**Upper**	**Sig**
**Indirect**	Smoking (no) ⇒ E-selectin ⇒ HOMA-IR	−1.55	−4.09	−0.13	*
	Smoking (no) ⇒ CRP ⇒ HOMA-IR	−0.04	−1.25	1.19	
	Sex (females) ⇒ E-selectin ⇒ HOMA-IR	2.40	0.46	5.80	*
	Sex (females) ⇒ CRP ⇒ HOMA-IR	0.09	−2.29	2.89	
	AGE ⇒ E-selectin ⇒ HOMA-IR	−1.11	−2.58	−0.27	*
	AGE ⇒ CRP ⇒ HOMA-IR	0.01	−0.42	0.64	
	HEI ⇒ E-selectin ⇒ HOMA-IR	−7.87	−17.48	−2.43	*
	HEI ⇒ CRP ⇒ HOMA-IR	−0.07	−3.38	1.98	
	HbA1c ⇒ E-selectin ⇒ HOMA-IR	9.26	3.40	18.66	*
	HbA1c ⇒ CRP ⇒ HOMA-IR	0.12	−3.49	3.47	
	Total/HDL Chol.⇒ E-selectin ⇒ HOMA-IR	1.92	0.34	4.49	*
	Total/HDL Chol.⇒ CRP ⇒ HOMA-IR	0.03	−0.89	1.13	
	BMI ⇒ E-selectin ⇒ HOMA-IR	2.72	0.97	5.54	*
	BMI ⇒ CRP ⇒ HOMA-IR	0.09	−2.23	2.70	
	WHR ⇒ E-selectin ⇒ HOMA-IR	5.59	−3.88	20.90	
	WHR ⇒ CRP ⇒ HOMA-IR	0.14	−4.18	7.67	
	Antidiabetic use (yes) ⇒ E-selectin ⇒ HOMA-IR	−0.60	−3.94	2.69	
	Antidiabetic use (yes) ⇒ CRP ⇒ HOMA-IR	−0.03	−1.73	0.99	
	Anti-hyperlipidemic use (yes)⇒ E-selectin ⇒ HOMA-IR	0.13	−1.95	2.25	
	Anti-hyperlipidemic use (yes)⇒ CRP ⇒ HOMA-IR	−0.06	−2.04	1.57	
	Anti-hypertensive use (no) ⇒ E-selectin ⇒ HOMA-IR	−2.39	−5.25	−0.70	*
	Anti-hypertensive use (no) ⇒ CRP ⇒ HOMA-IR	−0.01	−0.69	0.46	
	Number of MET (min/week) ⇒ E-selectin ⇒ HOMA-IR	0.62	−0.02	1.93	
	Number of MET (min/week) ⇒ CRP ⇒ HOMA-IR	0.00	−0.23	0.19	
**Component**	Smoking (no) ⇒ E-selectin	−2.88	−5.81	−0.03	*
	E-selectin ⇒ HOMA-IR	5.57	2.34	9.02	*
	Smoking (no) ⇒ CRP	−1.04	−1.97	−0.17	*
	CRP ⇒ HOMA-IR	0.39	−9.27	12.18	
	Sex (females) ⇒ E-selectin	4.38	0.45	8.34	*
	Sex (females) ⇒ CRP	2.30	1.13	3.54	*
	AGE ⇒ E-selectin	−0.21	−0.36	−0.06	*
	AGE ⇒ CRP	0.04	−0.01	0.09	
	HEI ⇒ E-selectin	−1.51	−2.80	−0.44	*
	HEI ⇒ CRP	−0.17	−0.43	0.11	
	HbA1c ⇒ E-selectin	5.45	3.19	8.00	*
	HbA1c ⇒ CRP	1.01	0.30	1.71	*
	Total/HDL Chol.⇒ E-selectin	1.75	0.12	3.40	*
	Total/HDL Chol. ⇒ CRP	0.40	−0.0004	0.84	
	BMI ⇒ E-selectin	0.50	0.24	0.78	*
	BMI ⇒ CRP	0.23	0.13	0.34	*
	WHR ⇒ E-selectin	10.03	−9.07	28.60	
	WHR ⇒ CRP	3.60	−2.28	9.39	
	Anti-diabetic use (yes) ⇒ E-selectin	−1.11	−6.47	4.92	
	Anti-diabetic use (yes) ⇒ CRP	−0.87	−2.57	1.17	
	Anti-hyperlipidemic use (yes) ⇒ E-selectin	0.24	−3.32	3.81	
	Anti-hyperlipidemic use (yes) ⇒ CRP	−1.57	−2.60	−0.54	*
	Anti-hypertensive use (no) ⇒ E-selectin	−4.46	−7.75	−1.29	*
	Anti-hypertensive use (no) ⇒ CRP	−0.23	−1.21	0.73	
	Number of MET (min/week) ⇒ E-selectin	0.0011	−0.0001	0.0028	
	Number of MET (min/week) ⇒ CRP	−0.00005	−0.0003	0.0003	
**Direct**	Smoking (no) ⇒ HOMA-IR	−0.43	−9.57	9.72	
	Sex (females) ⇒ HOMA-IR	8.97	−3.07	24.96	
	AGE ⇒ HOMA-IR	−2.01	−6.92	3.25	
	HEI ⇒ HOMA-IR	7.23	−25.98	62.75	
	HbA1c ⇒ HOMA-IR	86.19	46.82	144.03	*
	Total/HDL Chol. ⇒ HOMA-IR	30.81	17.62	45.21	*
	BMI ⇒ HOMA-IR	48.68	34.63	64.94	*
	WHR ⇒ HOMA-IR	13.21	5.44	21.85	*
	Anti-diabetic use (yes) ⇒ HOMA-IR	34.10	10.21	66.90	*
	Anti-hyperlipidemic use (yes) ⇒ HOMA-IR	18.97	6.95	32.92	*
	Anti-hypertensive use (no) ⇒ HOMA-IR	2.37	−8.45	14.41	
	Number of MET (min/week) ⇒ HOMA-IR	−1.47	−4.58	1.21	
**Total**	Smoking (no) ⇒ HOMA-IR	−2.01	−11.03	8.22	
	Sex (females) ⇒ HOMA-IR	11.69	−1.08	27.63	
	AGE ⇒ HOMA-IR	−3.09	−8.10	2.08	
	HEI ⇒ HOMA-IR	−1.28	−31.94	47.04	
	HbA1c ⇒ HOMA-IR	103.68	61.31	170.45	*
	Total/HDL Chol. ⇒ HOMA-IR	33.37	19.69	47.93	*
	BMI ⇒ HOMA-IR	52.86	38.84	68.63	*
	WHR ⇒ HOMA-IR	13.84	5.73	22.43	*
	Anti-diabetic use (yes) ⇒ HOMA-IR	33.25	8.62	65.71	*
	Anti-hyperlipidemic use (yes) ⇒ HOMA-IR	19.05	7.45	34.00	*
	Anti-hypertensive use (no) ⇒ HOMA-IR	−0.08	−10.64	11.31	
	Number of MET (min/week) ⇒ HOMA-IR	−0.86	−4.02	2.15	

† only for total, direct, and indirect paths. Percentages reported per 10 years increase in age, 10-units increase in PA, 0.1-unit increase in WHR, 2-units increase in total/HDL ratio, 3% increase in HbA1c, 10 kg/m^2^ increase in BMI, 1,000 number of MET (min/week). Under component paths, 10-units increase in CRP, and 10-units increase in E-selectin.

‡ CIs computed with method: Bias corrected bootstrap (3,000 repl.). N = 685.

* *P* < 0.05.

AGE, Age in years; BMI, body mass index; CIs, confidence intervals; CRP, C-reactive protein; HbA1c, glycated hemoglobin A; HEI, healthy eating index; HOMA-IR, homeostatic model assessment of IR; MET, metabolic equivalent of task; WHR, waist-hip ratio; HDL: High-density lipoprotein;

Unexpectedly, WHR was strongly associated with increased HOMA-IR (%CHIR = 13.84; 95% CI: [5.73, 22.43]), but this effect was not mediated by either CRP or E-selectin. The use of anti-hyperlipidemic or anti-diabetic medications was associated with significantly elevated HOMA-IR, likely reflecting the underlying diabetes and hyperlipidemia, which were confirmed contributors to increased HOMA-IR in our study. CRP did not mediate the relationship between any of the covariates and HOMA-IR.

## Discussion

Our analysis suggested that E-selectin had indirect-only mediation for the positive association between female sex and HOMA-IR, as indicated by significant indirect effect. This finding highlights the increased vulnerability of postmenopausal women to endothelial dysfunction and subsequent IR. Consistent with our results, a multi-ethnic cohort study by Song et al. [23] reported that higher E-selectin levels were associated with an increased risk of diabetes in postmenopausal women. However, studies directly comparing sex-specific differences in this context are lacking, underscoring the novelty of our findings and the need for further research on the underlying pathophysiology.

We also found that E-selectin partially mediates the positive associations between HbA1c, BMI, dyslipidemia (assessed via total/HDL cholesterol ratio), and IR. Elevated BMI and its associated inflammatory state promote endothelial activation, resulting in higher soluble E-selectin levels. Previous studies have confirmed a significant positive correlation between serum E-selectin and BMI [17], and weight loss has been shown to reduce E-selectin levels [24, 25]. Additionally, increased E-selectin is positively associated with HbA1c in diabetic patients within the first 2 years of diagnosis, suggesting that metabolic inflammation associated with poor glycemic control contributes to endothelial activation and may impair β-cell function [26]. The elevation of free fatty acids in response to these metabolic alterations and the resulting inflammatory response impair the phosphatidylinositol 3-kinase (PI3K)–dependent insulin signaling pathway in endothelial cells. This disruption reduces NO production and blunts NO-mediated vasodilation and blood flow [27]. Blunting NO activity results in oxidative stress that activates endothelial cells and increases E-selectin expression [28]. Therefore, an increase in E-selectin reflects an ongoing decrease in insulin sensitivity.

Interventions targeting weight reduction and glycemic control reduce E-selectin levels, partially by mitigating oxidative stress on the endothelium [25]. Collectively, these findings support the concept that elevated BMI and dysregulated glycemia exacerbate endothelial inflammation, leading to increased E-selectin expression, impaired β-cell function, and reduced insulin sensitivity. Prior studies have also observed higher HOMA-IR in response to elevated E-selectin and CRP [29]. Our mediation results clarify which metabolic factors exert their effects on IR through endothelial damage and those acting independently of CRP.

Other factors—including age, absence of regular smoking, and healthier dietary habits—were suggested to be mediated by E-selectin for their inverse associations with HOMA-IR under indirect pathways. Previous research has shown that adherence to healthy dietary patterns, such as the Mediterranean diet, is associated with lower circulating E-selectin levels and improved insulin sensitivity [30, 31]. Similarly, smokers exhibit higher E-selectin levels than non-smokers, supporting our finding that smoking cessation protects vascular health and enhances insulin sensitivity [32]. Regarding age, our results align with prior work indicating that E-selectin levels decline with advancing age in healthy individuals [33]. To our knowledge, our study is the first to demonstrate that this inverse association between age and E-selectin may translate into improved IR, despite the total effect of age on HOMA-IR not reaching statistical significance.

Several studies, consistent with our findings, have reported a significant association between WHR and IR [34, 35]. Although elevated BMI and metabolic syndrome have been linked to higher CRP levels in Asian and non-Asian populations [36], our findings indicate that the association between WHR and IR is not mediated by either CRP or E-selectin. This suggests that other inflammatory markers—such as IL-6 [37], interleukin-1β (IL-1β) [38], TNF-α [39], or other proinflammatory biomarkers—may mediate this association.

As seen, CRP did not have a mediation effect for any of the associations between metabolic factors and IR. This can be explained by many factors. CRP is synthesized primarily in liver hepatocytes as a part of inflammatory cascade that is not related directly to IR but rather to systemic inflammation [40]. In mediation analysis, this relationship would not result in a significant indirect effect of metabolic factors on IR through CRP, underscoring that CRP's role is independent of IR.

Our findings underscore the pivotal role of endothelial dysfunction, as reflected by elevated E-selectin levels, in the pathogenesis of IR. Positioning E-selectin as a candidate biomarker for vascular-metabolic risk screening, can significantly impact the field of metabolic research. Vascular complications of diabetes have a large burden, and the use of screening bio-markers may be a novel and important step in prevention and management efforts.

Lifestyle modifications—including improved dietary habits [41], smoking cessation [42], and weight reduction [43]—are well-established strategies to enhance insulin sensitivity and cardiovascular health. Beyond lifestyle interventions, therapeutic approaches targeting endothelial dysfunction may further improve E-selectin levels and IR. For example, knockdown of nucleophosmin (NPM) has been shown to deactivate NF-κB downstream genes, including E-selectin, reducing atherosclerotic plaque formation [44]. Pharmacologically, statins (e.g., simvastatin) significantly reduce serum E-selectin, reflecting improved endothelial function [45]. Additionally, angiotensin-converting enzyme (ACE) inhibitors and angiotensin II receptor blockers (ARBs), such as irbesartan, have been associated with decreased soluble E-selectin levels [46, 47]. While these agents are primarily used for hypertension, growing evidence supports their beneficial effects in managing IR, particularly ACE inhibitors [48, 49].

The use of advanced statistical approaches, such as mediation and moderated-mediation analyses, is crucial for elucidating the role of inflammatory markers in the pathway linking metabolic factors to IR. Recent studies have applied similar methods to evaluate the mediation roles of leukocytes and lymphocytes in cardiometabolic and glycemic indices, demonstrating the value of these approaches for understanding disease pathophysiology [50].

The use of a cross-sectional design in our analysis may be a limitation, given that mediation analysis assumes a temporal sequence between the independent variable, mediator, and outcome. However, in our sample of older adults, the independent metabolic factors are likely to have preceded changes in E-selectin levels, based on established pathophysiological mechanisms reported in the literature.

In addition, having a US sample may limit the generalizability of our results. Additionally, using HOMA-IR as a proxy measure for IR could be viewed as a limitation, since it is not considered the gold standard method for assessing IR.

## Conclusion

In summary, our study demonstrates that E-selectin, but not CRP, partially mediates the associations between BMI, HbA1c, and total/HDL cholesterol ratio and IR in older adults. E-selectin also mediates (indirect-only mediation) inverse associations between age, healthy eating habits, and absence of smoking history and IR. WHR was directly associated with IR but without mediation by E-selectin, and CRP did not mediate the associations between any of the examined risk factors and IR. These results highlight the important role of endothelial dysfunction in metabolic regulation and indicate that E-selectin may serve as a promising biomarker for IR. Moreover, endothelial dysfunction should be considered in the development of future therapeutic strategies. Additional studies are warranted to explore the potential of targeted interventions in both clinical and experimental settings.
